# What preconception health services would the public find when searching the internet in Australia?: results from a simulated internet-search study

**DOI:** 10.1186/s12913-024-10559-2

**Published:** 2024-01-17

**Authors:** Amie Steel, Harriet Gibson, Jon Adams, Danielle Schoenaker

**Affiliations:** 1 School of Public Health, Faculty of Health, University of Technology Sydney, Ultimo, NSW Australia; 2 School of Primary Care, Population Sciences and Medical Education, Faculty of Medicine, University of Southampton, Southampton, UK; 3 NIHR Southampton Biomedical Research Centre, University of Southampton and University Hospital Southampton NHS Foundation Trust, Southampton, UK

**Keywords:** Preconception care, Health services, Healthcare access, Health literacy

## Abstract

**Background:**

Australian preventive health strategy outlines the importance of preconception health in improving health in the community, across multiple generations and places primary and community healthcare services as a central pillar to effective preconception care. However, there is no national implementation plan to see preconception care proactively offered in healthcare settings in Australia. Instead, there is evidence that most women search the internet for information about pregnancy planning and preparation. In response, this study explores the availability and characteristics of health services found by searching for preconception care online in Australia.

**Method:**

Simulated Google searches were conducted using search terms ‘preconception’ and the name of a city/town with a population > 50,000. Related terms, ‘fertility’ and ‘pregnancy’ were also searched. Characteristics of the health services and the information available on relevant websites were extracted and reported descriptively.

**Results:**

The searches identified 831 website links, including 430 websites for health services. The health services were most often located in cities/towns with populations equal to or less than 200 000 (54.2%), and housing multiple health professionals (69.8%) including a specialist doctor (66.5%), nurse (20.9%), psychologist/counsellor (2.0%) and/or naturopath (13.0%). All the health services identified online explicitly mentioned women among their target populations, while 69.1% (*n* = 297) also referred to providing services for men or partners. More than one third of websites included blogs (36.9%) while external links were included in 10.8% of the online sites.

**Conclusions:**

This study provides a preliminary examination of health services that may be found through internet-based searching by Australian consumers seeking health advice or support prior to becoming pregnant. Our descriptive results suggest couples may find a variety of health professionals when seeking health services for preconception care. Future research involving co-design of search terms with consumers, ongoing monitoring of health services and ensuring access to meaningful, and accurate information found through internet-searching are all necessary to ensure people of reproductive age are able to access the preconception health information and care they need.

**Supplementary Information:**

The online version contains supplementary material available at 10.1186/s12913-024-10559-2.

## Background

Preconception health and healthcare influence the chances of an intended and healthy pregnancy, as well as the immediate and future health and wellbeing of parents and their children [1]. While most women seek and receive care once pregnant, such care alone often does not enable meaningful reduction of pre-existing risks factors to achieve the best possible health outcomes for women and their babies [1]. There is now ample evidence that minimising risk factors prior to pregnancy, including health behaviours, management of medical conditions, environmental exposures and their wider social and economic determinants before pregnancy, represents an opportunity to reduce maternal and infant mortality and morbidity and prevent non-communicable disease in parents and their offspring [1, 2].

When viewed from a life course perspective, preconception health is relevant from an early age when biological capacity to become pregnant is developed and health behaviours are formed during childhood and adolescence [3]. Preconception care—defined as the provision of biomedical, behavioural and social health interventions to optimise the health of prospective parents [4]—is however, most relevant for individuals of reproductive age, when pregnancy is physically possible and preconception health information and intervention may become relevant [3]. While high-quality evidence from intervention studies investigating the health benefits of preconception care is scarce, evidence from developmental biology and large epidemiological studies calls for increased efforts to support people of reproductive age to prepare for pregnancy [1–3].

The importance of optimising preconception health is recognised in national and international policies and clinical guidelines [5–8]. These advocate for continued efforts to improve the health of the population more broadly and provide guidance on providing preconception care for individuals planning pregnancy. In Australia, both the 2020–2030 National Health Strategies for women and for men outline key priorities and actions to improve preconception health [5, 6]. These relate to improved awareness and preventive health promotion activity, as well as access to preconception-related information and healthcare services. This includes general health information and support for all people of reproductive age, as well as more targeted information and support for people planning pregnancy, including those with pre-existing conditions and fertility problems. Despite the scientific evidence and policy interest in preconception health and care, there has been only modest progress in implementing identified priorities and actions into practice.

The Australian National Health Strategies for women and men recognise the important role primary and community healthcare services play in their implementation [5, 6]. The multidisciplinary teams of healthcare professionals offering these services are commonly involved in providing preconception care, including general practitioners, specialist doctors, nurses, midwives and allied health professionals [9, 10]. While awareness of the concept and importance of preconception health, and of the opportunity to receive preconception care through health services, has been reported as generally limited among people of reproductive age [11, 12], this is likely to increase as a result of interventions being developed to meet the goals set out in the National Health Strategies.

With heightened awareness, people of reproductive age will increasingly search for information about preconception health and care. The internet is frequently used by people, including by couples from all socioeconomic levels, to gain health-related information, including about getting pregnant [13–15]. In Australia, 91% of the total population are active internet users [16] therefore online information can be a powerful tool to support education on preconception health and increase awareness about the availability of and access to preconception care.

The aims of this study were to identify the websites of Australian health services that consumers may find when searching for preconception services in their local area, and to describe the characteristics of these health services and the information they provide.

## Methods

### Design

This observational study employed an exploratory approach to identify the websites of health services appearing through simulated web searches on Google using search terms that may be commonly used to search for health services that provide information, advice and/or care related to preconception. The study methodology was informed by similar research searching the internet for preconception care information in Italy to assess its accuracy [17]. This previously used methodology was adapted using specific Australian locations in the search terms to suit the Australian geographical spread and the research question, as described in more detail below.

### Search strings selection

The search strings were developed based on the most common search terms identified based on previous Italian research by Agricola et al. [17], the research team’s knowledge of preconception health and care, and the population spread in Australia. As a result, three different searches were conducted for each city or town in Australia with more than 50 000 population (*n* = 28). The primary search string used ‘preconception’ combined with the name of the city or town, while additional search strings used ‘fertility’ or ‘pregnancy’ and the name of the city or town. The selection of these search terms was informed by the results from the Italian study and our research teams’ knowledge of the preconception landscape in Australia. ‘Pregnancy’ was selected as the most common search term identified by women [17]. We selected ‘preconception’ as the first general search term provided by health professionals (excluding ‘toxoplasmosis’ and ‘genetic screening’ which we deemed to be too specific to address this study’s aim). ‘Fertility’ was selected by the research team as a health issue commonly conflated with preconception care in Australia policy and practice. This approach was taken as there is no known consumer-based research in Australia that explores preferred language and terms for the preconception period. Locations were included in the search string as the study aimed to identify the health services individuals would find through internet searching, rather than general preconception health information. A full list of the locations included in the searches and their population size is provided in Supplementary File 1.

### Search selection of websites

We launched 124 searches on Google using each of the search strings between 29th April and 18th June 2021. The first search engine result page, containing up to 10 website links excluding sponsored websites, was saved on a hard drive for each search string.

### Data collection

A data extraction spreadsheet was created in Microsoft Excel. The items were developed based on the research team’s preconception health and care knowledge and the results of previous Australian and international research examining relevant consumer perspectives and behaviours, including preferred internet search terms [9, 10, 17–20]. The spreadsheet was then tested for each search string in one location and refined to ensure it allowed meaningful data to be collected on characteristics of the health services and the information they provide.

#### Data collection items

The data extraction spreadsheet was used by a researcher (HG) to collect the data resulting from each search string. Initially, for each search, each webpage was assessed to determine whether it was either associated with a health service or were another type of webpage. A description of the ‘other’ webpages was recorded but only the webpages identified as connected to a health service were further evaluated. Characteristics of health service webpages were documented based on whether the service related to a multi-professional clinic or a specific health professional. The primary health condition focus of the website was categorised (e.g., *preconception, infertility, pregnancy, general health*) based on the messaging contained in the content of the website landing page. If the recorded weblink directed the researcher to a secondary webpage, then the researcher navigated to the home page prior to allocating it to a category. The health service was categorised as ‘infertility’ if it mentioned providing or supporting fertility treatments such as in vitro fertilisation. The health service was categorised as ‘preconception’ if the website information indicated services distinct from fertility treatments that aimed to prepare either parent for a healthy pregnancy, birth and baby including into the child’s later life. ‘Pregnancy’ health services were categorised as websites that referred to providing care to women once they are pregnant. In addition to the primary health condition focus of the service, any reference to specific health care needs – *infertility care*, *preconception care*, *pregnancy care*, *postnatal care*, and *other* - was recorded as binary variables on separate items to allow multiple healthcare needs to be identified. The researcher also recorded the health professionals listed as practicing at the health service, informed by health professionals most commonly referenced in the literature regarding preconception care in Australia and elsewhere [9, 10]. These were general practitioner, specialist doctor, nurse or nurse practitioner, midwife, naturopath, acupuncturist, and other. The researcher also recorded whether the health service explicitly referred to providing services for men, women, or other specific populations. The websites were further examined to determine whether they provided information through blogs and/or links to external websites. External websites were categorised as research (e.g., links to peer-reviewed publications or other research sources), organisations (e.g., government agencies, non-government organisations), and other. The details (e.g., URL and webpage title) associated with any external links were also recorded.

### Statistical analysis

The data were exported into Stata 16.1 (StataCorp LLC) for analysis. Prior to analysis, the data were checked for missing data and discrepancies, and an additional variable was added to allow multiple hits from websites with the same root URL to be identified. ‘Other’ responses were also interrogated, and new variables were generated for all health professional categories captured as ‘other’. A new variable was generated for each of the following: *manual therapists* (e.g., chiropractor, physiotherapist, osteopath, massage therapist), *educators* (e.g., childbirth educator, doula, hypnobirthing trainer), *scientist* (e.g., embryologist, scientist, geneticist), *yoga/pilates* (i.e., yoga, pilates), *counsellor/psychologist* (e.g., counsellor, psychologist, social worker, psychotherapist), *dietician*, and *other ingestive therapies* (e.g., herbalist, homeopath, clinical nutritionist). A new variable was created to count the number of different health conditions or stages indicated as an area of focus on the identified websites, and an additional variable was generated to determine the websites that referenced providing support to individuals across all stages of the reproductive period (preconception/fertility, pregnancy, postnatal).

Descriptive frequencies and percentages were then generated for all variables and chi-square tests were conducted to identify the characteristics most associated with the websites identified by each category of searches (i.e., ‘preconception’, ‘pregnancy’, ‘fertility’), and for websites that were found to include information blogs or external links. The findings of the bivariate analysis were tested utilising a chi square test with effect size as determined by Cramer’s V. The effect size was classified as negligible association (0.00 and under 0.10); weak association (0.10 and under 0.20); moderate association (0.20 and under 0.40); relatively strong association (0.40 and under 0.60); strong association (0.60 and under 0.80) and very strong association (0.80 and under 1.00), as reported by Rea and Parker [21].

## Results

The searches resulted in identification of 831 website links after removal of sponsored links, with a median of 27 (min 23, max 30) non-sponsored website links per location. Of these sites, 57.9% (*n* = 481) resulted from a search using a locality with a population equal to or less than 200 000, and 51.7% (*n* = 430) were websites associated with a health service. A list of all identified health service websites is provided in Supplementary File 2. The number of health service website links identified through the searches was greatest for search strings using the term ‘fertility’ (*n* = 194) and less common for ‘preconception’ (*n* = 133) and ‘pregnancy’ (*n* = 103).

Table 1 presents the characteristics of websites associated with health services identified through the searches. The websites from all searches were most frequently associated with localities with populations equal to or less than 200 000 (54.2%). Most health services housed multiple health professionals (69.8%), including a specialist doctor (66.5%), nurse (20.9%), psychologist/counsellor (20.0%), naturopath (13.0%), midwife (12.3%) and manual therapist (10.7%). The website information indicated the services primarily focused on infertility (44.2%), and this was even more frequent when each website’s healthcare focus was counted independently (70.6%). Preconception care (60.8%) and pregnancy care (51.8%) were also identified for more than half of the websites. The highest proportion of websites (*n* = 172, 40.1%) included reference to focusing on two of the four healthcare categories (infertility, preconception, pregnancy, postnatal care) with less indicating the service provided care for one (*n* = 108, 25.2%), three (*n* = 53, 12.4%) or all four (*n* = 94, 21.9%) categories and 32.8% (*n* = 141) providing support across preconception/fertility, pregnancy and postnatal periods (data not shown in Table 1). All services explicitly mentioned women among the populations targeted through their services, while 69.1% (*n* = 297) also referred to providing services for men and partners. Information pages and blogs were included in 36.9% of identified webpages while external links were included in 10.8%.

**Table 1:** Characteristics of websites promoting health services for preconception, fertility, and pregnancy, compared with other health services’ websites identified using the study search protocol (*n* = 430)

	All	Preconception (*n* = 133)			Fertility (*n* = 194)			Pregnancy (*n* = 103)		
	n (%)	n (%)	p*	V	n (%)	p*	V	n (%)	p*	V
Locality										
* Population equal to or less than 200 000*	233 (54.2)	82 (61.7)	0.04	0.01	111 (57.2)	0.3	-	40 (38.8)	< 0.001	0.01
* Population greater than 200 000*	197 (45.8)	51 (38.4)			83 (42.8)			63 (61.2)		
Service type										
* Multi-professional clinic*	300 (69.8)	73 (54.9)	< 0.001	0.22	155 (79.9)	< 0.001	0.20	72 (69.9)	0.9	-
* Specific health professional*	130 (30.2)	60 (45.1)			39 (20.1)			31 (30.1)		
Primary health condition focus based on homepage content										
* Infertility*	190 (44.2)	45 (33.8)	< 0.001	0.26	128 (66.0)	< 0.001	0.48	17 (16.5)	< 0.001	0.43
* General health*	99 (23.0)	52 (39.1)			20 (10.3)			27 (26.2)		
* Pregnancy*	99 (23.0)	26 (19.6)			19 (9.8)			54 (52.4)		
* Preconception*	37 (8.6)	8 (6.0)			26 (13.4)			3 (2.9)		
* Other*	5 (1.2)	2 (1.5)			1 (0.5)			2 (1.9)		
Health professionals working at the health service‡										
*Specialist doctor*	286 (66.5)	66 (49.6)	< 0.001	0.24	169 (87.1)	< 0.001	0.40	51 (49.5)	< 0.001	0.20
*Nurse*	90 (20.9)	15 (11.3)	0.001	0.16	54 (27.8)	0.001	0.16	21 (20.4)	0.9	
*Psychologists/Counsellors*	86 (20.0)	29 (21.8)	0.5	-	48 (24.7)	0.03	0.11	9 (8.7)	0.001	0.16
*Naturopath*	56 (13.0)	39 (29.3)	< 0.001	0.32	15 (7.7)	0.003	0.14	2 (1.9)	< 0.001	0.18
*Midwife*	53 (12.3)	9 (6.8)	0.02	0.11	10 (5.2)	< 0.001	0.20	34 (33.0)	< 0.001	0.35
*Manual therapist* ^‡^	46 (10.7)	18 (13.5)	0.2	-	2 (1.0)	< 0.001	0.28	26 (25.2)	< 0.001	0.26
*Scientist* ^‡^	41 (9.5)	11 (8.3)	0.6	-	30 (15.5)	< 0.001	0.18	0 (0.0)	< 0.001	0.18
*Acupuncturist*	33 (7.7)	12 (9.0)	0.5	-	17 (8.8)	0.4	-	4 (3.9)	0.1	-
*General practitioner*	24 (5.6)	10 (7.5)	0.2	-	7 (3.6)	0.1	-	7 (6.8)	0.5	-
*Educator* ^‡^	21 (4.9)	5 (3.8)	0.5	-	9 (4.6)	0.8	-	7 (6.8)	0.3	-
*Other ingestive therapies*	20 (4.7)	14 (10.5)	< 0.001	0.19	5 (2.6)	0.06	-	1 (1.0)	0.04	0.03
*Dietitian*	16 (3.7)	6 (4.5)	0.5	-	5 (2.6)	0.3	-	5 (4.9)	0.5	-
*Yoga/pilates*	9 (2.1)	3 (2.3)	0.9	-	1 (0.5)	0.04	0.10	5 (4.9)	0.03	0.11
Target population of the health service										
*Women*	430 (100.0)	133 (100.0)	-	-	194 (100.0)	-	-	103 (100.0)	-	-
*Men*	297 (69.1)	93 (69.9)	0.8	-	152 (78.4)	< 0.0001	0.18	52 (50.5)	< 0.001	0.26
*Other (e.g., children, illness populations)*	95 (22.1)	42 (31.6)	0.002	0.15	38 (19.6)	0.3		15 (14.6)	0.04	0.10
Healthcare needs addressed at the health service										
*Infertility care*	303 (70.6)	89 (67.4)	0.3	-	186 (95.9)	< 0.001	0.50	28 (27.2)	< 0.001	0.53
*Preconception care*	261 (60.8)	99 (75.0)	< 0.001	0.19	127 (65.5)	0.08	-	35 (34.0)	< 0.001	0.31
*Pregnancy care*	222 (51.8)	87 (65.9)	< 0.001	0.19	45 (23.2)	< 0.001	0.52	90 (87.4)	< 0.001	0.41
*Postnatal care*	201 (46.9)	84 (63.6)	< 0.001	0.22	42 (21.7)	< 0.001	0.46	75 (72.8)	< 0.001	0.29
*Other care (e.g., for specific health conditions)*	161 (37.5)	73 (55.3)	< 0.001	0.25	40 (20.6)	< 0.001	0.32	48 (46.6)	0.03	0.10
*Healthcare across preconception, pregnancy, and postnatal life stages*	141 (32.8)	70 (52.6)	< 0.001	0.28	41 (21.1)	< 0.001	0.23	30 (29.1)	0.4	-
Website information sources										
Blogs	157 (36.9)	49 (37.4)	0.8	-	81 (41.8)	0.05	0.10	27 (26.2)	0.01	0.12
Links to external sources	46 (10.8)	10 (7.6)	0.2	-	21 (10.9)	0.9	-	15 (14.6)	0.2	-
*Link to research publication or website*	6 (1.4)	1 (0.8)	0.5	-	2 (1.0)	0.6	-	3 (2.9)	0.1	-
*Link to external government or non-government organisation*	46 (10.8)	10 (7.6)	0.2	-	20 (10.4)	0.8	-	16 (15.5)	0.07	-
*Link to other external source*	5 (1.2)	3 (2.3)	0.2	-	1 (0.5)	0.3	-	1 (1.0)	0.8	-

‡ Professions included in categories as follows: *manual therapists -* chiropractor, physiotherapist, osteopath, massage therapist; *educators* - childbirth educator, doula, hypnobirthing trainer; *scientist -* embryologist, scientist, geneticist; *yoga/pilates -* yoga, pilates; *counsellor/psychologist* - counsellor, psychologist, social worker, psychotherapist; o*ther ingestive therapies* - herbalist, homeopath, clinical nutritionist

*p value from chi-square test comparing each category with the other two categories (for example, comparing the ‘preconception’ category with the ‘fertility’ and ‘pregnancy’ categories combined)

V = Cramer’s V reporting effect size defined as negligible association (0.00 and under 0.10); weak association (0.10 and under 0.20); moderate association (0.20 and under 0.40); relatively strong association (0.40 and under 0.60); strong association (0.60 and under 0.80) and very strong association (0.80 and under 1.00). This is only reported if *p* value is < 0.05

When compared to health service websites identified using search strings including ‘fertility’ and ‘pregnancy’, the websites found through searching ‘preconception’ had a greater frequency of websites linked to services for specific health professionals (V = 0.22) and of identifying general health as the primary health condition focus for the health service (V = 0.26). These health services’ websites listed naturopaths (V = 0.32) and other ingestive therapies (e.g., herbalist, homeopath, clinical nutritionist) (V = 0.19) more frequently, and specialist doctors (V = 0.24), nurses (V = 0.16) and midwives (V = 0.11) less frequently than other websites. They also had a higher rate of listing other target populations such as children or specific illness populations (V = 0.15), and of providing services across the preconception, pregnancy and postnatal life stages (V = 0.28) as well as other specific health conditions (V = 0.28).

Websites found through searches using ‘fertility’ had a much greater frequency of primarily focusing on ‘infertility’ within the health service (V = 0.48) and listing a specialist doctor (V = 0.40) among the health care team, compared with websites found when searching for ‘preconception’ or ‘pregnancy’. These websites also had a lower rate of identifying pregnancy (V = 0.52) or postnatal (V = 0.46) care or support for other health conditions (V = 0.32) through the health service.

The health service websites identified through use of ‘pregnancy’ as a search term had a relatively strong association with the primary health condition focus of the health service (V = 0.43) and a greater frequency of listing a midwife (V = 0.35) or manual therapist (V = 0.26) among the health service’s providers when compared with websites found when searching for ‘preconception’ or ‘fertility’. They also had a lower frequency of identifying men and partners among their target population (V = 0.26) and of providing infertility care (V = 0.53) or preconception care (V = 0.31) but a greater frequency of listing postnatal care (V = 0.29).

## Discussion

This study provides a preliminary examination of health services that can be found through internet-based searching by Australian consumers seeking health advice or support prior to becoming pregnant. Our simulated searches found 481 websites that provide information on health services across Australia that support pregnancy planning and preparation. Characteristics of these services varied based on the search terms used, including ‘preconception’, ‘fertility’ and ‘pregnancy’. While specific details of these differences are important, the overall significance of these findings are also relevant. In particular, the overall results highlight the lack of preconception-focused health services identified through searches using the ‘pregnancy’ – women’s preferred search term to find preconception information as identified by previous research [17] - and suggests a misalignment between the terminology used by health professionals and consumers. However, future research should verify the preferred search terms used by Australians seeking preconception information to verify this gap.

The services found through the internet searches were more commonly multi-professional clinics. Importantly, the health professionals practicing through these clinics sometimes included providers beyond those commonly considered as involved in preconception and maternity care support (e.g., naturopaths, massage therapists), particularly for individuals who may not be experiencing fertility issues. It is possible that this finding arose due to the broadness of the search terms used and the need for multidisciplinary clinics to rely on equally broad terms when describing their website services. However, due to the lack of clarity regarding who is ultimately responsible for preconception care [9] it is possible that consumers may not know to search for specific types of health professionals when seeking preconception services. While specialist doctors are undoubtedly central to contemporary fertility treatments [22], preconception health extends beyond assistance with becoming pregnant and includes changes in health behaviours to improve the overall health of both parents with the aim of achieving a healthier pregnancy and improving short- and long-term health outcomes of the baby [22]. Individuals seeking preconception care require access to health care that can provide holistic support and address the preconception risk factors known to impact on maternal and child outcomes; including, but not limited to, dietary intake, lifestyle behaviours, psychosocial factors, and infectious disease [22]. While preconception care is acknowledged within Australia’s women’s and men’s health strategies [5, 6], there is no nationally coordinated implementation plan and there appears to be a notable lack of clarity regarding who should be providing such care [9]. As such, the finding that health professionals such as naturopaths are practicing in almost one third of all services listed through searches for ‘preconception’ is notable and aligns with previous research reporting women who are attempting to conceive are more likely to consult with a naturopath compared to women not attempting to conceive [10]. Importantly, while Australian naturopaths commonly report a special interest in women’s health, frequently discuss common preconception risk factors with their patients (e.g., diet, lifestyle, alcohol intake, environmental exposures), and recommend diet and lifestyle changes to their patients [23], little is known about the content of those discussions or their alignment with the evidence-base regarding preconception health and care. However, it is also worth noting that there are similar gaps regarding the content and evidence-base of other health professionals’ patient communication about such topics [9, 24, 25]. Further research investigating the health literacy and preconception health practice behaviours of all Australian primary and community-based healthcare professionals is needed to fill these gaps and inform the implementation of preconception health care that optimises use of the health workforce.

Our study also found moderate to relatively strong associations between the search term used and the primary health condition focus of the services linked to the identified websites. However, the nature of this association appears somewhat complex. For example, there is a higher frequency of preconception as a primary focus among services identified through use of ‘fertility’ as a search term compared to ‘preconception’. Instead, ‘preconception’ health service websites more commonly focused on general health or pregnancy. The reason for this difference is unknown but may be related to preconception care being more associated with general health and wellness rather than solely-focused on treatment of fertility issues [4, 26]. As such it may be that the websites identified through using ‘fertility’ as a search term are listing preconception services within the specific context of improved fertility while health services associated with other websites may employ a broader lens within their preconception care approach. However, these possible interpretations need to be tested through additional research. A further complexity regarding available health services relates to continuity across reproductive health care. Specifically, websites identified through the ‘fertility’ search had a lower rate of listing care provided across preconception, pregnancy and postnatal life stages, while ‘preconception’ search results identified care provided across all reproductive life stages in more than half of the identified health service websites. This finding helps identify a novel area for research into continuity of care for reproductive health. It queries how continuity may be provided to couples planning a family from preconception through the postnatal (or interconception) period; an identified priority in maternity care albeit with a focus on antenatal and intrapartum services [27, 28]. While preconception-to-postnatal continuity is not contingent on one health service providing care throughout all reproductive health stages, preliminary stakeholder-engaged research has identified within-service and between-service continuity as a significant gap within Australia’s health system [29]. For this reason, the degree to which available services ensure continuity for couples by facilitating effective transition of care from and to other relevant health services and providers would benefit from closer attention.

Our study also found that men were less commonly included than women among the populations explicitly targeted in the content listed in the websites. There was also a weak association with the frequency of men and partners as a target population among websites found through use of ‘infertility’ (greater frequency) and ‘pregnancy’ (lesser frequency) as a search term. While men may be argued to have a lesser role in pregnancy, it is unclear why they are listed more commonly on ‘infertility’ health service websites than those found through ‘preconception’ searches and not with the same frequency as women in any of the searches. This finding is particularly important within the context of both fertility and preconception care as the health of both reproductive partners impacts the ability to conceive [30] and the health of the baby at birth and later in life [31, 32]. Notably, male-sexed reproductive partners contribute important diverse preconception health risk factors through, for example, their age [33], tobacco use [34–36], body weight [37], and occupational exposures [36], which may increase the risk of adverse offspring outcomes at birth (e.g., premature birth [33, 37], low birth weight [33, 37], macrosomia [37], stillbirth [37], congenital anomalies [37]) and during childhood (e.g., childhood cancer [34, 35], autism spectrum disorders [38], asthma [36]). Importantly, in some cases there may be a greater risk of adverse outcomes for men than when women have the same preconception exposure, with multigenerational epidemiological research finding father’s smoking before conception predicted their child’s early-onset asthma whereas mother’s smoking before conception did not [36]. Despite the importance of men’s preconception health, evidence suggests men and partners feel excluded from preconception and antenatal care and are viewed as ‘outsiders’ by health professionals in this context [39]. Furthermore, Australian general practitioners have reported perceived barriers to disseminating preconception health information to men including feeling uncertain about their own knowledge about men’s preconception health needs [40]. These potential barriers may be further exacerbated by lower rates of health care access [41] and greater incidence of risk taking behaviours [42] among male populations. Despite these challenges, evidence does suggest men may be receptive to preconception health counselling [43], and the reasons for their omission from between 20% and 30% of health service websites identified through use of ‘preconception’ and ‘infertility’ as a search term requires urgent attention.

A preliminary analysis of the consumer information on the identified websites found one third of the websites published additional information on pages with one in ten websites providing consumers with links to external websites for further information. This finding highlights the potential variability in publicly available preconception health information available in Australia. While there are some organisations that focus on health information for couples attempting pregnancy, such as the Your Fertility website produced via the Fertility Coalition [44], their primary focus is on fertility issues and as such may not be viewed by consumers as relevant for individuals seeking more general preconception health information. It is important for health promotion initiatives to carefully identify their audiences and develop messages that are audience-specific, particularly for mass communication methods such as websites [45]. As such, the content contained in external websites may not provide the information nor use the language that individuals seeking general preconception health information feel is meaningful or relevant to them [12]. The value of these external links may also be affected if the available information is inconsistent or does not align with the best available evidence. Indeed, Italian research suggests that the likelihood of finding online preconception care information that is consistent with clinical preconception guidelines is low [17]. A closer examination of the content of the health information contained on websites identified through internet searches for preconception, fertility and pregnancy care in Australia is urgently needed, alongside consumer involvement to ensure the information and messages are meaningful and relevant. Further research should also explore how the use of online information can be optimised to increase consumer awareness and uptake of preconception care.

### Limitations

The study methodology was informed by similar research that used simulated Google searches to find available preconception care information in Italy [17]. Our study is the first exploratory investigation of health services that may be found through internet-searching by consumers in Australia to support them to prepare for pregnancy. However, the searches were undertaken with single key word search terms, and these search terms were not developed through co-design with consumers and therefore the findings may deviate from the results of real-world internet searches. Future research should engage with the community to better understand the search terms they would use to find preconception health services and, should this vary significantly from the terms used in this study, additional internet search studies should be undertaken. It is also important to acknowledge that some health services, such as general practice, may not have a strong online presence under the assumption that it is the main entry point to the health care for most individuals. However, given the lack of clear responsibility for preconception care in the health system coupled with low awareness in the general community, this low online presence of general practice may result in individuals seeking preconception health advice and care from other healthcare services. Further research could also collect more detailed data, for example on the healthcare professionals involved in providing preconception care, which increasingly involves for example pharmacists, maternal and child health nurses and social prescribers. As the study was limited to locations with populations greater than 50,000 the findings may not be generalisable to other regional or remote communities with lower population levels. Furthermore, our findings provide a cross-sectional picture of the availability and characteristics of health services found by searching for preconception care in Australia, and repeated searches would be needed to monitor changes and improvements over time.

## Conclusions

The internet provides one increasingly important channel for those searching for information about preconception health and care and online information can be a powerful tool to support education on preconception health and increase awareness about the availability of and access to preconception care services. The ongoing availability and quality of such online information are issues that will only gain further significance for those seeking preconception care as well as all engaged in wider preconception practice, service provision and policy development. In the absence of a coordinated, government-led plan to implement preconception care in the Australian health system, community-led health services will arise that will need a strong online presence to provide evidence-based information and signposting to relevant local services.

### Electronic supplementary material

Below is the link to the electronic supplementary material.

Supplementary Material 1

Supplementary Material 2

## Data Availability

All data generated or analysed during this study are included in this published article [and its supplementary information files].

## References

[1] Stephenson J, Heslehurst N, Hall J, Schoenaker DA, Hutchinson J, Cade JE, Poston L, Barrett G, Crozier SR, Barker M (2018). Before the beginning: nutrition and lifestyle in the preconception period and its importance for future health. The Lancet.

[2] Fleming TP, Watkins AJ, Velazquez MA, Mathers JC, Prentice AM, Stephenson J, Barker M, Saffery R, Yajnik CS, Eckert JJ (2018). Origins of lifetime health around the time of conception: causes and consequences. The Lancet.

[3] Barker M, Dombrowski SU, Colbourn T, Fall CH, Kriznik NM, Lawrence WT, Norris SA, Ngaiza G, Patel D, Skordis-Worrall J (2018). Intervention strategies to improve nutrition and health behaviours before conception. The Lancet.

[4] World Health Organization. : Meeting to develop a global consensus on preconception care to reduce maternal and childhood mortality and morbidity: World Health Organization Headquarters, Geneva, 6–7 February 2012: Meeting report. 2013.

[5] Department of Health. National Women’s Health Strategy 2020–2030. Australian Government; 2019. https://bit.ly/3HKuj7U.

[6] Department of Health (2019). National men’s Health Strategy 2020–2030. In.

[7] Schoenaker D, Stephenson J, Connolly A, Shillaker S, Fishburn S, Barker M, Godfrey K, Alwan NA, UK Preconception Partnership (2022). Characterising and monitoring preconception health in England: a review of national population-level indicators and core data sources. J Dev Orig Health Dis.

[8] Dorney E, Boyle J, Walker R, Hammarberg K, Musgrave L, Schoenaker D, Jack B, Black KI (2022). A systematic review of clinical guidelines for Preconception Care. Semin Reprod Med.

[9] Steel A, Lucke J, Reid R, Adams J (2016). A systematic review of women’s and health professional’s attitudes and experience of preconception care service delivery. Fam Pract.

[10] Steel A, Adams J, Sibbritt D (2017). The characteristics of women who use complementary Medicine while attempting to Conceive: results from a nationally Representative Sample of 13,224 Australian women. Women’s Health Issues.

[11] Poels M, Koster MPH, Boeije HR, Franx A, van Stel HF (2016). Why do women not use preconception care? A systematic review on barriers and facilitators. Obstet Gynecol Surv.

[12] Schoenaker D, Gafari O, Taylor E, Hall J, Barker C, Jones B, Alwan NA, Stephenson J. Informing public health messages and strategies to raise awarness of pre-conception health: a public consultation. The Lancet 2021, 398.

[13] Bass S (2003). How will internet use affect the patient? A review of computer network and closed internet-based system studies and the implications in understanding how the use of the internet affects patient populations. J Health Psychol.

[14] Weissman A, Gotlieb L, Ward S, Greenblatt E, Casper RF (2000). Use of the internet by infertile couples. Fertil Steril.

[15] Bunting L, Boivin J (2007). Decision-making about seeking medical advice in an internet sample of women trying to get pregnant. Hum Reprod.

[16] Active internet users as. percentage of the total population in Australia from 2015–2022 [https://www.statista.com/statistics/680142/australia-internet-penetration/].

[17] Agricola E, Gesualdo F, Pandolfi E, Gonfiantini MV, Carloni E, Mastroiacovo P, Tozzi AE (2013). Does googling for preconception care result in information consistent with international guidelines: a comparison of information found by Italian women of childbearing age and health professionals. BMC Med Inf Decis Mak.

[18] Steel A, Adams J, Sibbritt D, Broom A, Gallois C, Frawley J (2012). Utilisation of complementary and alternative medicine (CAM) practitioners within maternity care provision: results from a nationally representative cohort study of 1,835 pregnant women. BMC Pregnancy Childbirth vol.

[19] Steel A, Lucke J, Adams J. The prevalence and nature of use of preconception services by women with chronic health conditions: an integrative review. BMC Women Health. 2015;15(14). 10.1186/s12905-12015-10165-12906. 10.1186/s12905-015-0165-6 PMC4338627 25783639

[20] Stephenson J, Patel D, Barrett G, Howden B, Copas A, Ojukwu O, Pandya P, Shawe J (2014). How do women prepare for pregnancy? Preconception experiences of women attending antenatal services and views of health professionals. PLoS ONE.

[21] Rea LM, Parker RA. Designing and conducting survey research: a comprehensive guide. John Wiley & Sons; 2014.

[22] Marino JL, Moore VM, Rumbold AR, Davies MJ (2011). Fertility treatments and the young women who use them: an Australian cohort study. Hum Reprod.

[23] Steel A, Schloss J, Leach M, Adams J (2020). The naturopathic profession in Australia: a secondary analysis of the Practitioner Research and Collaboration Initiative (PRACI). Complement Ther Clin Pract.

[24] Mazza D, Chapman A, Michie S (2013). Barriers to the implementation of preconception care guidelines as perceived by general practitioners: a qualitative study. BMC Health Serv Res.

[25] Ojukwu O, Patel D, Stephenson J, Howden B, Shawe J (2016). General practitioners’ knowledge, attitudes and views of providing preconception care: a qualitative investigation. Ups J Med Sci.

[26] Dorney E, Black KI (2018). Preconception care. Australian J Gen Pract.

[27] Jenkins MG, Ford JB, Todd AL, Forsyth R, Morris JM, Roberts CL (2015). Women׳ s views about maternity care: how do women conceptualise the process of continuity?. Midwifery.

[28] Sandall J, Soltani H, Gates S, Shennan A, Devane D. Midwife-led continuity models versus other models of care for childbearing women. Cochrane Libr 2016. 10.1002/14651858.CD004667.pub5 PMC8663203 27121907

[29] Steel A. Creating a Vision for Family-Centered Health Services for the Five ‘Trimesters’: Co-Design Meeting Summary Report. University of Technology Sydney; 2021. http://hdl.handle.net/10453/152294.

[30] Turner KA, Rambhatla A, Schon S, Agarwal A, Krawetz SA, Dupree JM, Avidor-Reiss T (2020). Male infertility is a women’s health issue—research and clinical evaluation of male infertility is needed. Cells.

[31] Steel A, Carter T (2021). Balancing our gaze on preconception health and care to include men. Adv Integr Med.

[32] Caut C, Schoenaker D, McIntyre E, Vilcins D, Gavine A, Steel A (2022). Relationships between women’s and men’s modifiable preconception risks and health behaviors and maternal and offspring health outcomes: an umbrella review. Semin Reprod Med.

[33] Khandwala YS, Baker VL, Shaw GM, Stevenson DK, Lu Y, Eisenberg ML. Association of paternal age with perinatal outcomes between 2007 and 2016 in the United States: population based cohort study. *bmj* 2018, 363. 10.1136/bmj.k4372 PMC6207919 30381468

[34] Liu R, Zhang L, McHale CM, Hammond SK. Paternal smoking and risk of childhood acute lymphoblastic leukemia: systematic review and meta-analysis. *Journal of oncology* 2011, 2011. 10.1155/2011/854584 PMC3132639 21765828

[35] Metayer C, Zhang L, Wiemels JL, Bartley K, Schiffman J, Ma X, Aldrich MC, Chang JS, Selvin S, Fu CH (2013). Tobacco smoke exposure and the risk of childhood acute lymphoblastic and myeloid leukemias by cytogenetic subtype. Cancer Epidemiol Prev Biomarkers.

[36] Svanes C, Koplin J, Skulstad SM, Johannessen A, Bertelsen RJ, Benediktsdottir B, Bråbäck L, Elie Carsin A, Dharmage S, Dratva J (2017). Father’s environment before conception and asthma risk in his children: a multi-generation analysis of the Respiratory Health in Northern Europe study. Int J Epidemiol.

[37] He Y, Xie X, Tang W, Ma X (2017). Maternal and paternal obesity and adverse pregnancy outcomes in China: a cohort study. The Lancet.

[38] Surén P, Gunnes N, Roth C, Bresnahan M, Hornig M, Hirtz D, Lie KK, Lipkin WI, Magnus P, Reichborn-Kjennerud T (2014). Parental obesity and risk of autism spectrum disorder. Pediatrics.

[39] Kotelchuck M, Lu M (2017). Father’s role in preconception health. Matern Child Health J.

[40] Hogg K, Rizio T, Manocha R, McLachlan RI, Hammarberg K (2019). Men’s preconception health care in Australian general practice: GPs’ knowledge, attitudes and behaviours. Aust J Prim Health.

[41] Sternberg P, Hubley J (2004). Evaluating men’s involvement as a strategy in sexual and reproductive health promotion. Health Promot Int.

[42] Wang Y, Hunt K, Nazareth I, Freemantle N, Petersen I. Do men consult less than women? An analysis of routinely collected UK general practice data. BMJ open 2013, 3(8). 10.1136/bmjopen-2013-003320 PMC3753483 23959757

[43] Shawe J, Patel D, Joy M, Howden B, Barrett G, Stephenson J (2019). Preparation for fatherhood: a survey of men’s preconception health knowledge and behaviour in England. PLoS ONE.

[44] Your, Fertility. [https://www.yourfertility.org.au/].

[45] Corcoran N. Communicating health: strategies for health promotion. Sage; 2013.

