# Supplementary material for: What preconception health services would the public find when searching the internet in Australia?: results from a simulated internet-search study
Source: BMC Health Serv Res. 2024 Jan 17;24:93. doi: 10.1186/s12913-024-10559-2 (PMC10795199; doi:10.1186/s12913-024-10559-2)
Supplement: Supplementary file 1 — Supplementary Material 1 [file 12913_2024_10559_MOESM1_ESM.docx]

Supplementary Table 1: Localities searched, by population size

| **Locality** | **State or Territory** | **Population size** |
| --- | --- | --- |
| [Sydney](https://en.wikipedia.org/wiki/Sydney) | [New South Wales](https://en.wikipedia.org/wiki/New_South_Wales) | 4,914,343 |
| [Melbourne](https://en.wikipedia.org/wiki/Melbourne) | [Victoria](https://en.wikipedia.org/wiki/Victoria_(Australia)) | 4,893,870 |
| [Brisbane](https://en.wikipedia.org/wiki/Brisbane) | [Queensland](https://en.wikipedia.org/wiki/Queensland) | 2,430,180 |
| [Perth](https://en.wikipedia.org/wiki/Perth_metropolitan_region) | [Western Australia](https://en.wikipedia.org/wiki/Western_Australia) | 2,045,479 |
| [Adelaide](https://en.wikipedia.org/wiki/Adelaide) | [South Australia](https://en.wikipedia.org/wiki/South_Australia) | 1,340,794 |
| Gold Coast–Tweed Heads | Queensland/New South Wales | 693,671 |
| Newcastle–Maitland | [New South Wales](https://en.wikipedia.org/wiki/New_South_Wales) | 491,474 |
| Canberra–Queanbeyan | Australian Capital Territory/New South Wales | 462,136 |
| [Sunshine Coast](https://en.wikipedia.org/wiki/Sunshine_Coast,_Queensland) | [Queensland](https://en.wikipedia.org/wiki/Queensland) | 341,069 |
| [Central Coast](https://en.wikipedia.org/wiki/Central_Coast_(New_South_Wales)) | [New South Wales](https://en.wikipedia.org/wiki/New_South_Wales) | 335,470 |
| [Wollongong](https://en.wikipedia.org/wiki/Wollongong) | [New South Wales](https://en.wikipedia.org/wiki/New_South_Wales) | 306,034 |
| [Geelong](https://en.wikipedia.org/wiki/Geelong) | [Victoria](https://en.wikipedia.org/wiki/Victoria_(Australia)) | 275,794 |
| [Hobart](https://en.wikipedia.org/wiki/Hobart) | [Tasmania](https://en.wikipedia.org/wiki/Tasmania) | 216,682 |
| [Townsville](https://en.wikipedia.org/wiki/Townsville) | [Queensland](https://en.wikipedia.org/wiki/Queensland) | 181,668 |
| [Cairns](https://en.wikipedia.org/wiki/Cairns) | [Queensland](https://en.wikipedia.org/wiki/Queensland) | 153,951 |
| [Toowoomba](https://en.wikipedia.org/wiki/Toowoomba) | [Queensland](https://en.wikipedia.org/wiki/Queensland) | 138,223 |
| [Darwin](https://en.wikipedia.org/wiki/Darwin,_Northern_Territory) | [Northern Territory](https://en.wikipedia.org/wiki/Northern_Territory) | 133,331 |
| [Ballarat](https://en.wikipedia.org/wiki/Ballarat) | [Victoria](https://en.wikipedia.org/wiki/Victoria_(Australia)) | 107,652 |
| [Bendigo](https://en.wikipedia.org/wiki/Bendigo) | [Victoria](https://en.wikipedia.org/wiki/Victoria_(Australia)) | 100,991 |
| Albury–Wodonga | New South Wales/Victoria | 94,837 |
| [Launceston](https://en.wikipedia.org/wiki/Launceston,_Tasmania) | [Tasmania](https://en.wikipedia.org/wiki/Tasmania) | 88,178 |
| [Mackay](https://en.wikipedia.org/wiki/Mackay,_Queensland) | [Queensland](https://en.wikipedia.org/wiki/Queensland) | 80,264 |
| [Rockhampton](https://en.wikipedia.org/wiki/Rockhampton) | [Queensland](https://en.wikipedia.org/wiki/Queensland) | 79,081 |
| [Bunbury](https://en.wikipedia.org/wiki/Bunbury,_Western_Australia) | [Western Australia](https://en.wikipedia.org/wiki/Western_Australia) | 74,591 |
| [Coffs Harbour](https://en.wikipedia.org/wiki/Coffs_Harbour) | [New South Wales](https://en.wikipedia.org/wiki/New_South_Wales) | 72,541 |
| [Melton](https://en.wikipedia.org/wiki/Melton,_Victoria) | [Victoria](https://en.wikipedia.org/wiki/Victoria_(Australia)) | 72,177 |
| [Bundaberg](https://en.wikipedia.org/wiki/Bundaberg) | [Queensland](https://en.wikipedia.org/wiki/Queensland) | 71,309 |
| [Wagga Wagga](https://en.wikipedia.org/wiki/Wagga_Wagga) | [New South Wales](https://en.wikipedia.org/wiki/New_South_Wales) | 56,675 |
| [Hervey Bay](https://en.wikipedia.org/wiki/Hervey_Bay) | [Queensland](https://en.wikipedia.org/wiki/Queensland) | 55,345 |
| Mildura–Wentworth | Victoria/New South Wales | 52,176 |
| Shepparton–Mooroopna | [Victoria](https://en.wikipedia.org/wiki/Victoria_(Australia)) | 52,104 |
